# Utilization of preventive care among migrants and non-migrants in Germany: results from the representative cross-sectional study ‘German health interview and examination survey for adults (DEGS1)’

**DOI:** 10.1186/s13690-021-00609-0

**Published:** 2021-05-24

**Authors:** Anne Starker, Claudia Hövener, Alexander Rommel

**Affiliations:** Department of Epidemiology and Health Monitoring, Robert Koch Institute, General Pape-Straße 62-66, 12101 Berlin, Germany

**Keywords:** Preventive health services, Health check, Dental health services, Cancer screening, Skin cancer, Cervical cancer, Emigration and immigration, Health survey

## Abstract

**Background:**

In Germany, different health checks for adults are offered for primary and secondary prevention. Previous findings indicate that preventive care utilization varies according to social determinants, especially migration background. This study examined the extent to which migration background is associated with preventive care utilization, independent of factors like age and socioeconomic status and whether length of stay in Germany has a positive effect on the use of preventive care.

**Methods:**

The first wave of the ‘German Health Interview and Examination Survey for Adults’ (DEGS1) is a comprehensive data collection facilitating the description of the utilization of general health checks, dental check-ups, skin cancer screening, and cervical cancer screening among people aged 18–79 years with and without migration background. Migration background was differentiated in first-generation migrants having immigrated to Germany themselves or second-generation migrants born in Germany. First-generation migrants were further differentiated by length of stay in Germany, and second-generation migrants as having one or two parents who were born abroad. Multivariate binary logistic regression models with average marginal effects were calculated to analyse the associations between preventive care utilization and migration background.

**Results:**

The sample comprised 7987 participants, 1091 of whom had a migration background. Compared with non-migrants, women and men with migration background— particularly first-generation migrants with length of stay <=20 years in Germany — make less use of preventive care. This association was observed statistically independent from sociodemographic factors. For dental check-ups a significantly lower use was also found for first-generation migrants who have lived in Germany for more than 20 years and second-generation of migrants with two parents born abroad. Post-model predictions showed that the utilization rates of first-generation migrants are gradually converging to the average values for non-migrants.

**Conclusions:**

Our findings suggest inequalities in realized access to preventive care for first-generation migrants particularly for those who have lived in Germany for 20 years or less. Barriers to the utilization of preventive care may be addressed by informing migrant communities about preventive health care services at an early stage after immigration using migrant-sensitive information strategies.

## Background

In Germany, different health checks for adults are offered for primary and secondary prevention. These include general health checks (GHCs), cancer screening examinations, and dental check-ups (DCUs). These health checks are available free of charge to women and men at certain ages through the statutory health insurance system. The main objectives of these health checks are to prevent diseases by reducing important risk factors and to detect diseases at a precursor or early stage to prevent disease progression or becoming chronic. More specifically, the purpose of the GHC is to identify and assess health risks and to detect major diseases such as cardiovascular diseases, type 2 diabetes mellitus, and kidney disease at an early stage, as well as to provide prevention-oriented counselling [1]. Similarly, DCUs serve to prevent oral and dental diseases or detect them at an early stage and to give educational advice regarding dental health [2]. The aim of early cancer detection measures is to avert potential risks to the health of eligible persons by thoroughly examining suspected cases, diagnosing cancer at an early stage, and, if necessary, providing timely cancer treatment [3].

Previous findings indicate that the utilization of preventive care varies according to social characteristics, such as age, sex, socioeconomic status (SES), and also migration background [4–6]. According to the German Federal Statistical Office, in 2018, 20.8 million people with a migration background live in Germany [7], whereby a migration background is given if either the person himself or at least one parent was not born with German citizenship. Because of the increasing numbers of people immigrating from other European countries, asylum seekers and refugees [8], the percentage of foreigners in the German population grew from 18.8% in 2009 to 25.5% in 2018 [7, 9]. About 6.1 million people with a migration background were born in Germany and have not experienced migration themselves, and about 13.5 million were born abroad and migrated to Germany [7].

The association between migration and health is complex [10], and previous studies have shown that health risks and resources differ for people with a migration background, compared with those without a migration background (non-migrants) [6]; this difference is also reflected in the utilization of the healthcare system [11, 12].

The reasons for the differences in health care utilization between migrants and non-migrants have not been fully explored. In addition to possible differences in health needs, preferences, and expectations different barriers to the utilization of preventive care have been discussed a potential explanation for differences in utilization [13–15]. This includes the absent or limited health insurance coverage, language barriers, discrimination and insufficient information about and access to preventive care among migrant population [16, 17]. However, many studies have demonstrated that the use of preventive care is generally associated with a medium and high SES [5, 18]. Thus, it could be argued that the on average lower SES of migrants compared to non-migrants may partly or fully explain a different utilization of preventive care in people with migration background [19]. In contrast, others have suggested that migration background may be an independent factor regardless of SES [6, 16].

In addition results of previous studies indicated that there are differences in utilization of preventive care within migrant populations depending on whether they are first-generation migrants who have immigrated to the host country themselves or second-generation migrants who were born in the host country and of whom either one or both parents has immigrated [12, 13, 20, 21]. Furthermore it is also important to differentiate first-generation migrants based on their length of stay (LOS) because previous findings have indicated that differences in the utilization of preventive care between migrants and non-migrants decrease as migrants’ LOS in the host country increases [13, 20, 21]. Due to the heterogeneity of the migrant population general statements are therefore of limited applicability. For this reason in epidemiologic studies and databases, an exact definition and operationalization of migration background is required [22].

In summary, there is evidence of inequalities in the utilization of preventive care between migrants and non-migrants. However, we do not know whether these inequalities are migration-specific or caused by the lower SES of people with migration background. Migrant-specific explanations can therefore only claim validity once the independent effect of migration background for the use of preventive care has been proven. Furthermore, no studies have examined the association between LOS in Germany and the use of preventive services in detail. The present study therefore aimed to investigate to what extent migration background is associated with the utilization of preventive care in Germany. First, we hypothesized that migration background is negatively associated with the utilization of preventive care, regardless of sociodemographic factors. Secondly, we hypothesized that, among first-generation migrants, utilization behaviour converges to the utilization patterns of non-migrants as the LOS increases, and thirdly that LOS has a positive effect on the utilization of preventive care independent of age and SES.

## Methods

### Data

The ‘German Health Interview and Examination Survey for Adults’ (DEGS) is a comprehensive data collection that allows the description of the utilization of preventive care among migrants and non-migrants [6]. The first wave of DEGS (DEGS1) is part of the German health monitoring conducted by the Robert Koch Institute and is based on two-stage stratified cluster sampling. Primary sampling units (PSUs) are sampled from a list of German communities, stratified by district, with a classification that considers urbanization, regional population density, and administrative borders. Because the DEGS1 was designed to include participants from the German National Health Interview and Examination Survey 1998 (GNHIES98), the DEGS1 PSU sample first selected a total of 120 PSUs previously sampled for the GNHIES98. Then, to remain representative at the population level, additional PSUs (*N* = 60) were sampled [23, 24]. In each newly added PSU, random samples of individuals, stratified by 10-year age groups, were drawn from the PSU’s local population registers [25]. Because participants in the GNHIES98 cohort had aged by 10–14 years when the DEGS1 sample was drawn, only completely newly recruited individuals were included in the DEGS1 sample for the 18–28 years age group. Persons aged 30 years and older were sampled from the GNHIES98 sample as necessary to replace the participants expected to be lost to follow-up. The numbers of newly sampled PSUs and individuals per PSU were determined by statistical power and sample size considerations [25]. In addition, oversampling by a factor of 1.5 was carried out for persons without German citizenship to compensate both for the lower participation rate and for the higher proportion of failed contacts in this group. The aim of this oversampling was to ensure that the proportion of foreigners among the participants corresponded to the proportion of foreigners in the population.

Fieldwork for the DEGS1 extended over 3 years, from 25 November 2008 to 26 November 2011. Two mobile study teams, consisting of specifically trained health professionals, successively visited each PSU. Data collection comprised interviews, examinations, and tests. Two different self-administered questionnaires were distributed to the 18 to 64 years and to the ≥65 years age groups to obtain information on physical, psychological, and social aspects of their health. Information on issues such as diagnoses or therapies was obtained by physicians via computer-assisted personal interviews. In addition to German, the questionnaires were available in Russian, Turkish, Serbo-Croatian, and English [23–25].

In total, 8152 individuals participated in the DEGS1: 4193 first-time participants (response rate: 42%) and 3959 revisited participants of the GNHIES98 (response rate: 62%). The average response rate was comparable to that of other European national health surveys [26]. A nonresponse analysis and comparison of selected indicators using data from census statistics indicated a high level of representation of the German residential population aged 18–79 years [23]. Because the DEGS1 sample was designed to be representative of the entire population in the 18–79 years age group, 165 original GNHIES98 participants aged 80 years or older at the time of the DEGS1 data collection were excluded from the analyses.

### Indicators (characteristics)

#### Outcome

Self-reported utilization of GHC, DCU, skin cancer screening (SCS), and cervical cancer screening (CCS) were the outcome measures of this study. In the self-administered questionnaire for the DEGS1 for participants aged 35 and over were asked whether they had ever attended a GHC (response options: ‘yes’, ‘no’, or ‘not sure’). Participants who answered this question affirmatively were then asked ‘Have you also taken part in a health check-up in the last 2 years?’ (‘yes’ or ‘no’). Participants up to the age of 64 were asked if they attended a DCU regularly, at least once per year (‘yes’ or ‘no’). In computer-assisted personal interviews, DEGS1 participants aged 35 and older were asked if they had ever had a full body skin examination (SCS) (‘yes’, ‘no’, or ‘not sure’). Participants who answered affirmatively were then asked about the date of the last examination, with possible response options: ‘within the last 12 months’, ‘within the last 2 years’, ‘within the last 3 years’, ‘within the last 10 years’, ‘more than 10 years ago’, and ‘not sure’. Women aged 20 years and older were asked about their use of CCS tests in the same way. Based on these answers we generated binary variables (yes = 1, no = 0) indicating GHC attendance in the last 2 years, DCU attendance in the last 12 months, SCS attendance in the last 2 years, and CCS attendance in the last 12 months. The classification follows guidelines for the various health checks applicable at the time of the DEGS1 study in 2011 (Table 1) [2, 4, 27, 28].

**Table 1:** Health checks for adults covered by statutory health insurance in Germany in 2011 (outcome measures)

Health check	Aim	Type of examination	Target group	Age	Examination interval
Dental Check-Up	Early detection of tooth, mouth and jaw diseases	Examination of dental condition and oral cavity	Women, men	From 18 years	Two examinations within 1 year
General Health Check-Up	Early detection of chronic diseases and their main risk factors	Anamnesis	Women, men	From 35 years	Every 2 years
		Physical examination			
		Laboratory tests (blood, urine)			
		Medical counselling			
Cancer Screening	Early detection of cervical cancer	Cervical smear test	Women	From 20 years	Annually
	Early detection of skin cancer	Whole-body skin examination	Women, men	From 35 years	Every 2 years

#### Determinants

When a participant or at least one of their parents was born abroad, the participant was considered to have a migration background (Table 2) [6, 29].

**Table 2:** Typology of migration background in DEGS1 study

			Country of birth of DEGS1 participant’s parents	
			one abroad	both
			one in Germany	abroad
**Country of birth of DEGS1 participant**	**abroad**	LOS in Germany > 20 years	**I**	
		LOS in Germany ≤ 20 years	**II**	
	**in Germany**		**III**	**IV**

**I** First-generation migrants with LOS in Germany > 20 years

**II** First-generation migrants with LOS in Germany <= 20 years

**III** Second-generation migrants with one-sided migration background

**IV** Second-generation migrants with two-sided migration background

*Abbreviations*: *LOS* length of stay, *DEGS1* first wave of the German Health Interview and Examination Survey for Adults

Participants who were born abroad were classified as first-generation migrants, whereas participants born in Germany to foreign-born parents were classified as second-generation migrants. Within the group of second-generation migrants, a further distinction was made between those with one parent born abroad (one-sided migration background) and those with both parents born abroad (two-sided migration background). First-generation migrants were further classified by LOS in Germany into two groups of similar size (LOS ≤ 20 years, LOS > 20 years). This distinction was rather data-driven with the aim of a clear cut-off with sufficiently large case numbers in both categories. To enable more fine-grained analyses in first-generation migrants LOS was additionally used as a continuous variable (LOS in years).

SES was determined using an index that included information on education and vocational training, professional status, and net household income (weighted by household needs); SES was classified as low, medium, or high [30].

### Statistical analysis

All analyses were conducted with Stata 15.1 (Stata Corp., College Station, TX, USA, 2017) using survey procedures for complex samples. This allowed to appropriately consider the clustering of the participants in sample points and to consider the weighting in the calculation of confidence intervals and *p*-values. Weighting factors were used to correct for deviations in the sample from the population structure in terms of age, sex, region, nationality, community type, and education level, and re-participation probability of the GNHIES98 participants.

Multivariate binary logistic regression was used to analyse the associations between the utilization of preventive care and its determinants. In addition to sex, age, and SES, the logistic regression models included first- and second-generation migration status, distinguishing between LOS ≤ 20 years and > 20 years for first-generation migrants and between one-sided and two-sided migration background for second-generation migrants. The analyses followed a block-wise modelling approach to quantify the extent to which the effect of single factors changed by adding further determinants. Since methodological studies have shown that the usual regression coefficients in logit and probit analyses cannot be compared between nested models average marginal effects (AMEs) were suggested as a procedure that allows valid direct comparison of effect sizes between different models [31, 32]. AMEs indicate the percentage increase in the probability of an event (dependent variable) when the independent variable changes by one unit [33]. The change in the AME between different models can be interpreted directly [31, 32]. Model 1 explored the extent to which migration background (four groups) was associated with the use of different types of preventive services (GHC, DCU, SCS, and CCS) controlling for age and sex (except for CCS, which is only relevant for women). Model 2 additionally controlled for SES. To illustrate the impact of LOS (as a continuous variable) on the utilization of preventive health services in first-generation migrants, we estimated the AME controlling for age, sex (except for CCS), and SES including interaction effects for sex, LOS and LOS squared (Model 3). To illustrate the effects identified in the models on the outcomes, adjusted utilization rates in percent of the population are presented as post-model predictions. These adjusted utilization rates are given by sex, fixing the effect of age at 50 years and the effect of SES at medium SES thus providing the sex-specific utilization of preventive care at representative values of age and SES [33].

## Results

The sociodemographic characteristics of the participants and the distribution of the utilization of preventive care in the study population are shown in Table 3. The sample comprised 7987 participants, 1091 of whom had a migration background. Second-generation migrants with both parents born abroad were the smallest group. Among all participants, preventive care attendance varied from 25.5% (SCS) to 77.2% (DCU).

**Table 3:** Sample characteristics

		n	% weighted	95% CI	
**Sex**	Female	4198	50.3	48.9	51.8
	Male	3789	49.7	48.2	51.1
**Age**	18–39 years	2086	33.5	32.4	34.6
	40–59 years	3131	39.7	38.6	40.7
	60+ years	2770	26.8	25.8	27.9
**Socioeconomic status**	Low	1238	19.7	18.3	21.3
	Medium	4743	60.3	58.7	61.8
	High	1916	20.0	18.5	21.6
	Missing	90			
**Migration background**	No	6595	80.4	78.3	82.3
	First-generation, LOS < = 20 years	277	6.4	5.4	7.6
	First-generation, LOS > 20 years	362	6.4	5.5	7.6
	Second-generation, two-sided	101	2.0	1.5	2.5
	Second-generation, one-sided	351	4.8	4.1	5.5
	Missing	301			
**General health check** within the last 2 years	Yes	1745	40.9	38.9	43.0
	No	2308	59.1	57.0	61.1
	Filtered (aged < 35 years)	3596			
	Missing	338			
**Dental check-up** at least once per year	Yes	4697	77.3	75.7	78.7
	No	1124	22.7	21.3	24.3
	Not asked (age 65+ years)	2049			
	Missing	117			
**Skin cancer screening** within the last 2 years	Yes	1667	25.5	23.9	27.2
	No	4448	74.5	72.8	76.1
	Filtered (aged < 35 years)	1547			
	Missing	325			
**Cervical cancer screening** within the last 12 months	Yes	2242	57.5	55.6	59.4
	No	1540	42.5	40.6	44.4
	Filtered (aged < 20 years or male)	3881			
	Missing	324			

*Source: DEGS1*

*Abbreviations*: *95% CI* 95% confidence interval, *LOS* length of stay

The multivariate analysis (*n* = 7987 after excluding cases with missing data) of the association between migration background and GHC utilization in the last 2 years showed a significant effect only for first-generation migrants with LOS < = 20 years. Compared with non-migrants, this group had a 21.2% lower probability of having attended a GHC in the last 2 years (Table 4, Model 1). After controlling for SES, the effect was reduced by 5.2% but remained statistically significant (Table 4, Model 2).

**Table 4:** Effect of migration background on preventive care utilization: binary logistic regression (average marginal effects)

		Model 1			Model 2			Loss in effect size^**a**^
		AME	95% CI		AME	95% CI		(%)
**General health check**								
Migration background	First-generation, LOS < = 20 years	**− 0.212**	− 0.294	− 0.130	**− 0.201**	− 0.286	− 0.115	−5.2
(Ref: No)	First-generation, LOS > 20 years	− 0.068	− 0.151	0.014	− 0.056	− 0.140	0.027	−17.6
	Second-generation, two-sided	− 0.113	− 0.387	0.160	− 0.095	− 0.375	0.186	−15.9
	Second-generation, one-sided	−0.019	− 0.099	0.060	− 0.015	− 0.095	0.065	−21.1
**Dental check-up**								
Migration background	First-generation, LOS < = 20 years	**− 0.236**	−0.303	− 0.168	**−0.204**	− 0.268	−0.140	−13.6
(Ref: No)	First-generation, LOS > 20 years	**− 0.134**	−0.216	− 0.051	**−0.103**	− 0.185	−0.022	−23.1
	Second-generation, two-sided	**−0.225**	−0.344	− 0.106	**−0.179**	− 0.289	−0.068	− 20.4
	Second-generation, one-sided	−0.037	−0.098	0.023	−0.034	− 0.095	0.027	−8.1
**Skin cancer screening**								
Migration background	First-generation, LOS < = 20 years	**−0.165**	−0.229	− 0.102	**−0.159**	− 0.225	−0.092	−3.6
(Ref: No)	First-generation, LOS > 20 years	− 0.047	−0.104	0.010	−0.031	− 0.091	0.029	− 34.0
	Second-generation, two-sided	−0.101	−0.222	0.019	−0.083	− 0.208	0.041	−17.8
	Second-generation, one-sided	0.013	−0.058	0.085	0.013	−0.058	0.083	0.0
**Cervical cancer screening**								
Migration background	First-generation, LOS < = 20 years	**−0.260**	−0.363	− 0.156	**−0.230**	− 0.327	−0.133	−11.5
(Ref: No)	First-generation, LOS > 20 years	− 0.080	−0.175	0.015	−0.066	− 0.164	0.032	−17.5
	Second-generation, two-sided	**−0.207**	−0.411	− 0.004	−0.153	− 0.348	0.042	−26.1
	Second-generation, one-sided	−0.037	−0.121	0.047	−0.037	− 0.122	0.048	0.0

*Source: DEGS1*

Model 1: controlled for age and sex; Model 2: controlled for age, sex and socioeconomic status;

Figures in bold indicate *p* < 0.05; ^a^These figures were calculated using the following formula: 100/AME Model 1 x (AME Model 2 - AME Model 1)

*Abbreviations*: *AME* average marginal effects, *95% CI* 95% confidence interval, *LOS* length of stay, *Ref* reference category

The results for the use of SCS within the last 2 years pointed in a similar direction, although the effect was slightly smaller: First-generation migrants with LOS < = 20 years had a 16.5% lower probability of using SCS, compared with non-migrants (Table 4, Model 1). After controlling for SES, the effect was reduced by 3.6% but remained statistically significant (Table 4, Model 2).

The findings were different for annual DCU attendance, where significant effects were found for first-generation migrants and for second-generation migrants with two-sided migration background. For first-generation migrants, significant effects that varied according to LOS were observed: Compared with non-migrants, the probability of DCU utilization was 23.6% lower for first-generation migrants with LOS < = 20 years and 13.4% lower for first-generation migrants with LOS > 20 years (Table 4, Model 1). The decline in effect size after controlling for SES in Model 2 was larger for the group with LOS > 20 years. For second-generation migrants with two-sided migration background, the effect was of the same order of magnitude as that observed for first-generation migrants with LOS < = 20 years (− 22.5%) (Table 4, Model 1), but with larger loss in effect size after controlling for SES (Table 4, Model 2).

For annual CCS attendance, significant results were visible for first-generation migrants with LOS < = 20 years and for second-generation migrants with two-sided migration background (Table 4, Model 1). The latter effect disappeared after additionally controlling for SES (Table 4, Model 2).

**Fig. 1:**
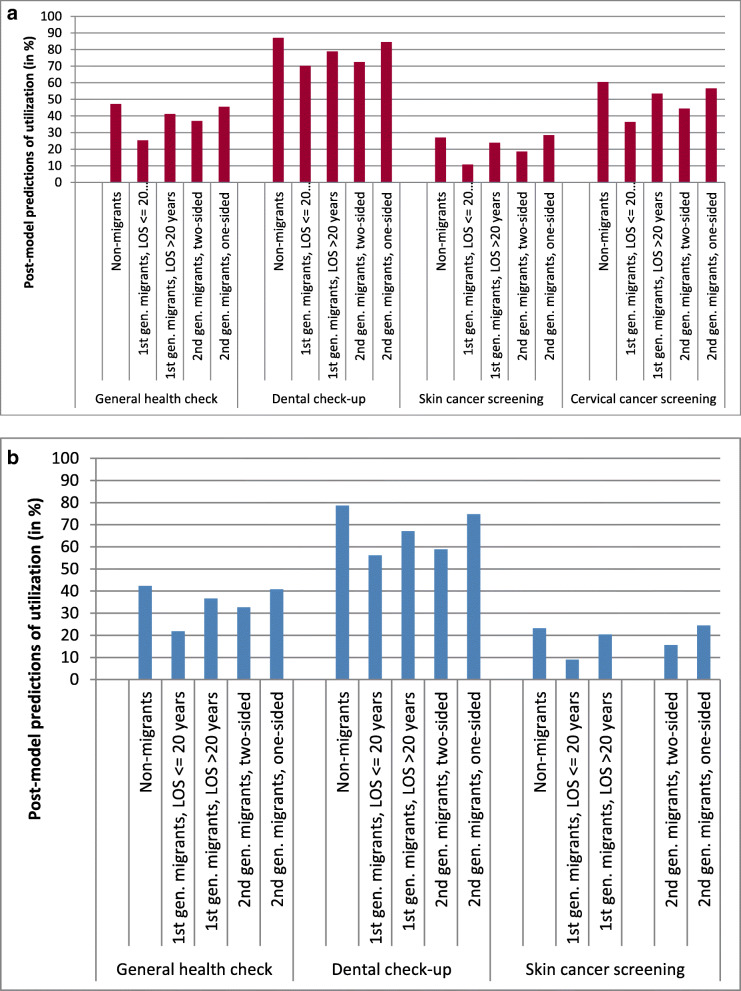
**a** Use of preventive health services in 50-year-old women with medium SES by migration background (model-based predictions based on model 2 expressed in utilization rates in %). *Source: DEGS1*. Abbreviations: 1st gen.: first generation, 2nd gen.: second generation, LOS: length of stay. **b** Use of preventive health services in 50-year-old men with medium SES by migration background (model-based predictions based on model 2 expressed in utilization rates in %). *Source: DEGS1*. Abbreviations: 1st gen.: first generation, 2nd gen.: second generation, LOS: length of stay

Figure 1a and b illustrate these findings with sex-specific post-model predictions based on model 2 results for respondents aged 50 years and with SES medium. Across the types of preventive care, the overall level of the predictions expressed in utilization rates in percent clearly differed, particularly between DCU and SCS. The results show that the prediction for preventive care services uptake was highest for women and men without a migration background. and lower among first-generation migrants with LOS < = 20 years. Based on model 2 this association was statistically significant (Table 4). Comparing women without a migration background and first-generation migrant women with LOS < = 20 years, the largest difference was found for SCS (2.5-times higher) and the smallest difference for CCS (1.2-times higher). For men, the largest difference was found for SCS (2.6-times higher), and the smallest difference for DCU (1.4-times higher). Furthermore, it is noticeable that for both sexes, for DCU, second-generation migrants with two-sided migration background also had a considerably lower utilization rate compared with non-migrants an association that approved to be statistically significant in model 2 (Table 4).

If the impact of LOS in years of first-generation migrants on the utilization of preventive health services is estimated the results suggest an increasing utilization with longer LOS with statistically significant effects for GHC and DCU but not for cancer screening (Table 5).

**Table 5:** Effect of length of stay (in years) in first-generation migrants on preventive care utilization: binary logistic regression (average marginal effects)

	Preventive health services	AME	95% CI	
Lengths of stay (in years)	General health check (GHC)	**0.006**	0.001	0.011
	Dental check-up (DCU)	**0.003**	0.000	0.006
	Skin cancer screening (SCS)	0.004	-0.002	0.009
	Cervic cancer screening (CCS)	0.005	-0.001	0.012

*Source: DEGS1*

Controlled for age, sex and socioeconomic status; Figures in bold indicate *p* < 0.05

*Abbreviations*: *AME* average marginal effects, *95% CI* 95% confidence interval

**Fig. 2:**
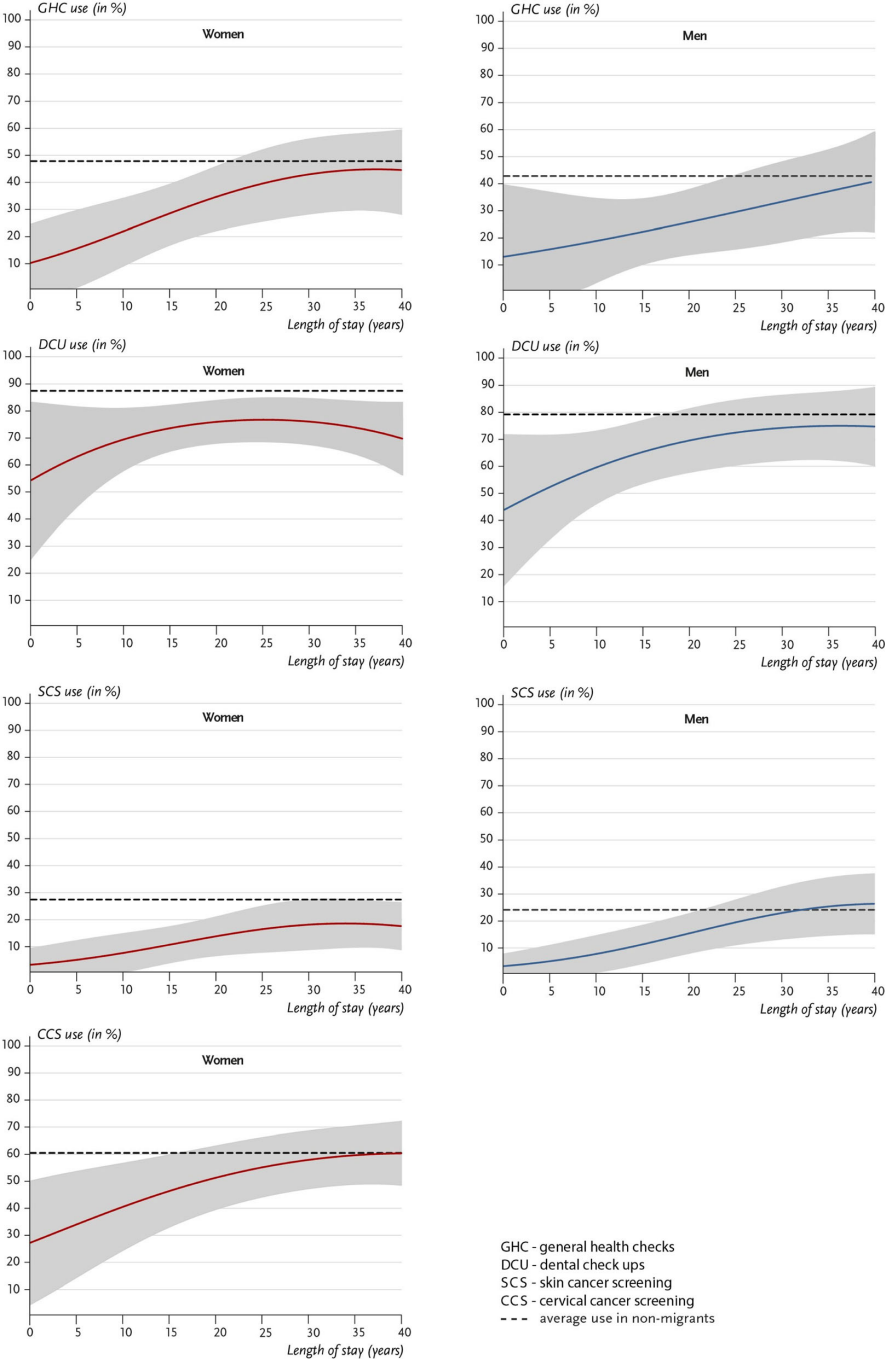
Use of preventive health services in first-generation migrants by length of stay and non-migrants (both expressed in utilization rates in % at age of 50 and medium SES; model-based predictions based on model 3 for migrants including interactions between sex and LOS and model 2 for non-migrants). *Source: DEGS1*

Expressed in predicted utilization rates in percent for respondents at an age of 50 and with medium SES the results illustrate these gradually converging utilization rates of first-generation migrants with increasing LOS to the average values for non-migrants (Fig. 2). However, the estimates also suggest that this is a long-term process. Assuming that this relationship continues to exist in similar strength under current conditions, full alignment can likely only be achieved after several decades.

## Discussion

This study examined to what extend migration background is associated with the utilization of preventive care. It became clear that a differentiation of first-generation migrants by LOS and second-generation migrants by one- or two-sided background enables a more precise analysis of the heterogeneous impact that migrant background may have on the outcome under study. We found that, compared with non-migrants, women and men with a migration background make less use of preventive care which applies particularly for first-generation migrants who have lived in Germany for 20 years or less. For this group, the hypothesis that migration background is negatively associated with the use of preventive care regardless of sociodemographic factors was clearly confirmed. For DCU, differences were also identified for first-generation migrants who have lived in Germany for more than 20 years and for second-generation migrants with two-sided migration background. After controlling for SES, we found a decline in the strength of the associations between having a migration background and lower utilization of preventive care. However, the association remains statistically significant. Moreover, independent of age and SES, longer LOS was associated with higher levels of the utilization of preventive care thus confirming the second and third hypothesis.

Previous studies in Germany showed significantly lower participation in the use of preventive care in the migrant population compared to people without a migration background for GHCs [34, 35], DCUs [34–37], and cancer screening examinations [6, 38]. This is consistent with our findings. However, these studies did not differentiate the migration status in more detail and thus disregard the heterogeneity of the migrant population.

The present study has shown that for second-generation migrants the distinction between a one-sided and a two-sided migration background is in part a relevant differentiation for the outcomes under study. Especially, the use of DCU was lower for migrants with a two-sided migration background compared to non-migrants, but not for migrants with a one-sided migration background. This insight is not possible when using a binary indicator for migration background. The few previous studies accounting for this factor have confirmed that people with a one-sided migration background and non-migrants are similar with respect to their health care utilization [16, 39].

Moreover, LOS is an important feature for further differentiating migration background in first-generation migrants, which is also confirmed by international studies [40–43]. In accordance with the present results, previous studies on migration and health considering LOS have shown that differences between migrants and non-migrants decrease as LOS increases (for CCS: [44, 45]; for DCU: [41]).

Although considerable inequalities in the utilization of preventive care have been observed between migrants and non-migrants (see [18] for an overview), the extent to which these differences are caused by the lower SES of people with a migration background has rarely been analysed [6, 16, 46]. In line with the present findings, previous studies examining this topic have shown no or only a slight decrease in the correlation between migration background and the use of preventive health care when controlling for socioeconomic factors. This supports the hypothesis that migration background is an independent determinant for the use of preventive health care services.

In our study, the findings for DCU differed markedly from the utilization patterns of GHC, SCS, and CCS in two respects. First, utilization rates for DCU were generally much higher compared with the other preventive services. This may be because, in Germany, regular prophylactic measures to maintain, promote, and improve the oral health of children and adolescents have been implemented as a regular service in nurseries and schools; obviously, many adults see regular outpatient visits to the dentist for preventive purposes as normal – even more since those who can prove regular dentist attendance receive an extra allowance (bonus) for dentures if required. Second, we found that the differences between non-migrants and migrants in DCU utilization persisted even among second-generation migrants with a two-sided migration background. Studies have shown that people with a migration background living in Germany have insufficient knowledge about the risk factors for caries (sugar consumption and inadequate oral hygiene) [37, 47]. It has also been shown that the use of oral health services is more complaint-oriented and less prevention-oriented among people with a migration background [48]. Moreover, having a low level of knowledge about the co-payment of caries prophylaxis has also been seen as a reason for low use of DCUs in people with migration background [49]. These knowledge gaps seem to be passed on to the next generation, an assumption that is supported by findings showing that children and adolescents with a migration backgrounds already have poorer oral health compared with their non-migrant counterparts in terms of use of DCUs and frequency of tooth brushing [50, 51].

This study entails the following limitations. First, because our study design was cross-sectional, causal statements cannot be made with certainty. However, the determinants under study (migration background, SES, age, and sex) precede service utilization and thus cannot be consequences of service utilization. Second, when interpreting the results, it should be noted that the data on service utilization are based on self-report, which may be prone to recall bias [40]. However, preventive services are taken up consciously and partly by invitation; thus, a high degree of accuracy in self-report data on the utilization of these services can be assumed. Third, individuals who were unable to provide written consent and those with a significant language barrier were excluded from participation in the DEGS1. Although foreign-language questionnaires were generally available, it was shown that better-integrated people with a migration background, namely second generation migrants and migrants with higher educational levels, were overrepresented in the DEGS1 sample [29] In part this bias was addressed in the analyses by adjusting for SES. Moreover, in DEGS1 nor it is possible to consider the country of origin of people with migrant background neither information on cultural or religious aspects was collected. Possible associations with the use of preventive care can therefore not be verified. When interpreting the results, it must be taken into account that the data collection for DEGS1 dates back about 10 years. In particular, immigration in the wake of the refugee crisis in 2015 has changed the size and composition of the migrant population in Germany in terms of age, education and country/culture of origin. Nevertheless, it can be assumed that the correlations found are still relevant, especially against the background of the increased need for support among newly arrived migrants in finding their way around the healthcare systems of the destination countries.

## Conclusions

Our findings suggest substantive inequities in realized access to preventive care for the first-generation migrants regardless of sociodemographic factors, particularly for those who have lived in Germany for 20 years or less. To reduce these inequities, barriers to accessing preventive care for people with a migration background should be addressed. For example, it could be promising to inform migrant communities about preventive health care services at an early stage after immigration using migrant-sensitive information strategies providing multilingual and diversity sensitive information about the preventive services in the German healthcare system and explaining how to access them. In organized cancer screening programmes with an invitation system like the German mammography screening programme or the early detection of colon and cervical cancer this could improve equal access to information about these services. Definitely, our finding suggest a general need for action regarding dental health, particularly with regard to the multilingual and diversity sensitive dissemination of knowledge about oral hygiene and tooth-friendly nutrition, but also on the use of preventive dental care.

## Data Availability

The ‘Health Monitoring’ Research Data Centre at the Robert Koch Institute (RKI) is accredited by the German Data Forum according to uniform and transparent standards (https://www.ratswd.de/en/data-infrastructure/rdc). The DEGS1 data set is freely accessible on application to interested scientists as de facto anonymized data for scientific secondary analysis. More detailed information on access, application forms and guidelines can be obtained from datennutzung@rki.de.

## Abbreviations

1st gen.: First generation
2nd gen.: Second generation
95% CI: 95% confidence interval
AME: Average marginal effect
CCS: Cervical cancer screening
DCU: Dental check-up
DEGS: German Health Interview and Examination Survey for Adults
DEGS1: First wave of German Health Interview and Examination Survey for Adults
GHC: General health check
GNHIES98: German National Health Interview and Examination Survey 1998
LOS: Length of stay
PSU: Primary sampling unit
SCS: Skin cancer screening
SES: Socioeconomic status
