# Correction: Utilization of preventive care among migrants and non-migrants in Germany: results from the representative cross-sectional study ‘German health interview and examination survey for adults (DEGS1)’

**DOI:** 10.1186/s13690-022-00992-2

**Published:** 2022-12-12

**Authors:** Anne Starker, Claudia Hövener, Alexander Rommel

**Affiliations:** 1 Department of Epidemiology and Health Monitoring, Robert Koch Institute, General Pape-Straße 62- 66, 12101 Berlin, Germany; 2 Charité – Universitätsmedizin Berlin, corporate member of Freie Universität Berlin and Humboldt-Universität zu Berlin, Institute for Public health, Charitéplatz 1, 10117 Berlin, Germany

**Correction: Arch Public Health 79, 86 (2021)**

**https://doi.org/10.1186/s13690-021-00609-0**

Following publication of the original article [1], the authors reported the second author affiliation needed to be added.

The correct author affiliations have been provided in this Correction.

The original article [1] has been corrected.
